# Chemically Induced Proximity for Targeted Protein *O-*GlcNAcylation

**DOI:** 10.1021/acs.accounts.6c00453

**Published:** 2026-09-30

**Authors:** Bowen Ma, Tongyang Xu, Zhihao Guo, Billy Wai-Lung Ng

**Affiliations:** † School of Pharmacy, Faculty of Medicine, 26451 The Chinese University of Hong Kong, Hong Kong; ‡ Li Ka Shing Institute of Health Sciences, Faculty of Medicine, 26451 The Chinese University of Hong Kong, Hong Kong; § CUHK-Hub of Obstetric and Paediatric Excellence, 26451 The Chinese University of Hong Kong, Hong Kong; ∥ Gerald Choa Neuroscience Institute, 26451 The Chinese University of Hong Kong, Hong Kong; ⊥ Peter Hung Pain Research Institute, 26451 The Chinese University of Hong Kong, Hong Kong

## Abstract

*O-*GlcNAcylation is a ubiquitous post-translational modification across the mammalian proteome, yet determining how individual *O-*GlcNAcylation events control specific protein functions remains a central challenge. The *O-*GlcNAc cycle is governed by two enzymes: *O-*GlcNAc transferase (OGT), which installs *O-*GlcNAc, and *O-*GlcNAcase (OGA), which removes it. Because these enzymes act on thousands of proteins, conventional methods, such as pharmacological inhibition of OGT or OGA, produce broad cellular effects that are difficult to assign to individual substrates. This gap has motivated the development of targeted *O-*GlcNAcylation strategies, including genetically encoded and chemical biology tools, which have shown that *O-*GlcNAc can be selectively installed on defined proteins to interrogate protein-specific *O-*GlcNAc functions.

This Account describes the development of *O-*GlcNAc TArgeting Chimeras, or OGTACs, as chemical tools for targeted *O-*GlcNAc installation. OGTACs are bifunctional molecules that can recruit OGT to a specific protein of interest. Specifically, an OGTAC consists of an OGT-engaging element, a linker, and a target-recognition motif, which together bring OGT into proximity with a selected substrate.

Our studies show that chemically induced proximity can redirect OGT toward selected proteins, thereby creating targeted *O-*GlcNAc writing that can partially bypass TPR-domain-mediated substrate recognition under engineered recruitment conditions. A central lesson is that productive *O-*GlcNAc writing requires not only recruitment, but also proper ternary-complex formation and noninhibitory OGT engagement. Moreover, the target-recognition motif determines the targetable protein space and achievable selectivity of the platform. By linking local *O-*GlcNAc installation to changes in protein turnover, signaling, and transcriptional output, OGTACs provide a conceptual framework for using induced proximity to modulate protein functions.

## Graphical abstract

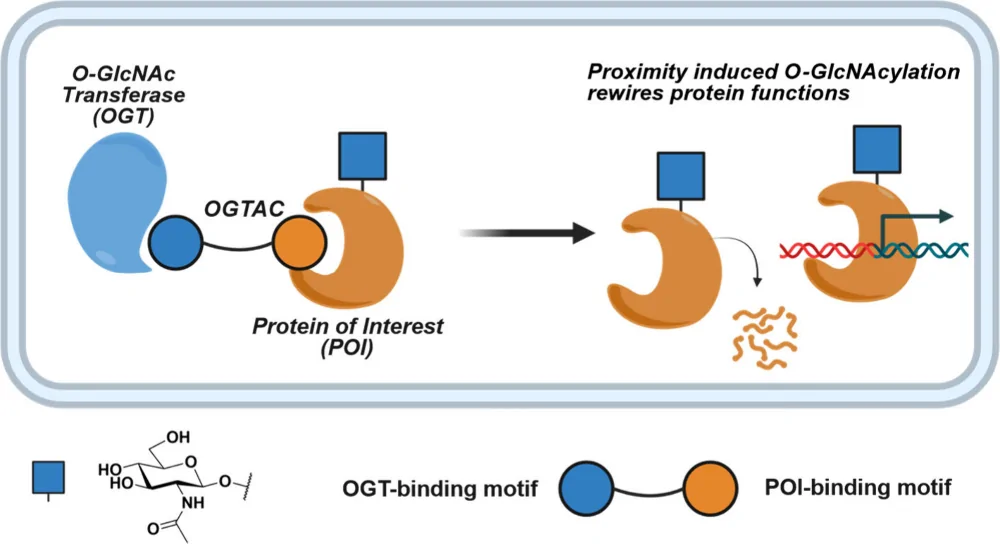

## Key References

-

Ma, B. ; Khan, K. S. ; Xu, T. ; Xeque Amada, J. ; Guo, Z. ; Huang, Y. ; Yan, Y. ; Lam, H. ; Cheng, A. S.-L. ; Ng, B. W.-L. Targeted Protein *O-*GlcNAcylation Using Bifunctional Small Molecules. J. Am. Chem. Soc. 2024,146, 9779–9789.10.1021/jacs.3c14380PMC1100994638561350.^1^ This study introduced chemogenetic OGTACs, establishing chemically induced proximity as a substrate-recognition mechanism for FKBP12^F36V^-OGT and revealing linker length, chemical reversibility, and OGT substrate-recognition domain truncation as key determinants of selective O-GlcNAc induction.
-

Guo, Z. ; Xu, T. ; Khan, K. S. ; Scheeff, S. ; Qin, Y. ; Yu, S.-Y. ; Lo, R. ; Li, Y. ; Xie, Y. ; Ma, B. ; Huang, Y. ; Yip, H. Y.-Y. ; Chung, C. Y.-S. ; Tamura, T. ; Hamachi, I. ; Ng, B. W.-L. Ligand-Directed Self-Assembling Chimeras for Targeted Protein *O-*GlcNAcylation. ACS Chem. Biol. 2025, 20, 2907–2916.10.1021/acschembio.5c00684PMC1272368241348108.^2^ This work addressed the challenge of recruiting OGT through its catalytic domain without suppressing its catalytic activity. Ligand-directed release chemistry and bioorthogonal self-assembly enabled transient OGT recognition while avoiding persistent active-site inhibition.
-

Xu, T. ; Guo, Z. ; Khan, K. S. ; Huang, Y. ; Ma, B. ; Ji, S. ; Liu, J. ; Felsher, D. W. ; Ng, B. W.-L. Targeted *O*-GlcNAcylation Enables Functional Rewiring of c-Myc. Cell Chem. Biol. 2026 10.1016/j.chembiol.2026.08.00842685690 (10.1016/j.chembiol.2026.08.008).^3^ This study extended the OGTAC concept by using an oligonucleotide as the target-recognition motif. Using a DNA-based oligo-OGTAC, this study supported a model in which OGT can be recruited to the oncogenic transcription factor, c-Myc, to alter its transcriptional outputs and cancer cell viability. It also illustrates the opportunities and specificity challenges of targeting transcription factors.
-

Xu, T. ; Ma, B. ; Li, Y. ; Guo, Z. ; Zhang, M. ; Ng, B. W.-L. Targeted *O-*GlcNAcylation of CK2α Triggers Its Ubiquitin-Proteasome Degradation and Alters Downstream Phosphorylation. ACS Chem. Biol. 2025, 20, 1646–1659.10.1021/acschembio.5c00223PMC1228148440521680.^4^ This study linked targeted O-GlcNAcylation to CK2α degradation and downstream phosphorylation rewiring, illustrating how OGTACs can uncover functional consequences of targeted O-GlcNAc installation.

## 1 Introduction

Unlike classical extracellular glycosylation, which typically involves complex, branched glycans, *O-*linked β-*N*-acetylglucosamine (*O-*GlcNAc) modification is a unique intracellular post-translational modification (PTM).^5,6^ It consists of a single monosaccharide dynamically attached to the serine, threonine, and potentially tyrosine residues^7^ on thousands of nuclear, cytosolic, and mitochondrial proteins.^8,9^ The entire cellular *O-*GlcNAc cycle is governed by just two highly conserved enzymes: *O-*GlcNAc transferase (OGT), which adds the sugar moiety, and *O-*GlcNAcase (OGA), which removes it (Figure 1A).^10,11^

**Figure 1:**
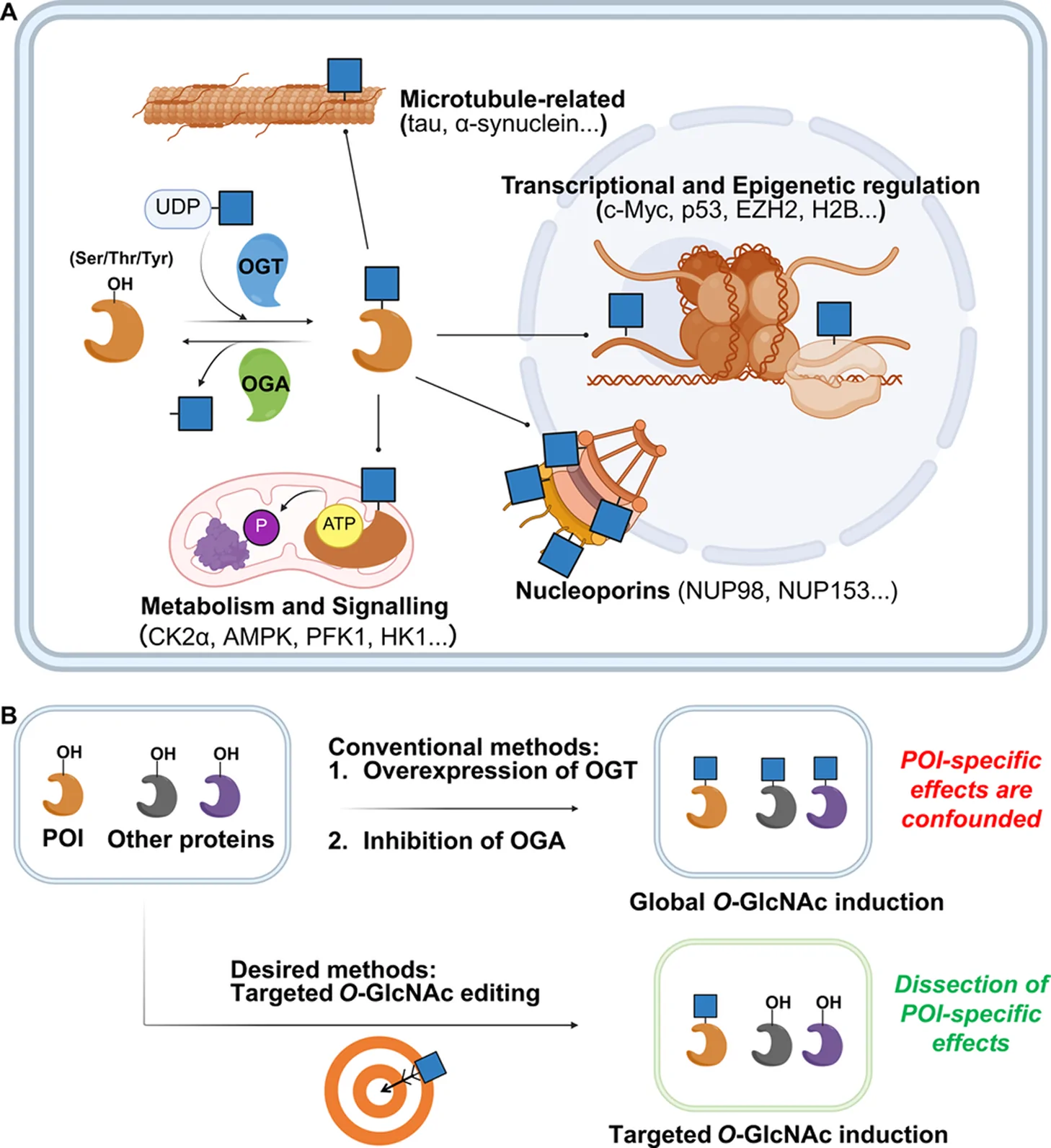
Global *O-*GlcNAc perturbation confounds assignment of protein-specific function. (A) *O-*GlcNAcylation regulates diverse intracellular processes, including metabolism, signaling, transcription, chromatin regulation, protein stability, and protein–protein interactions. (B) Conventional perturbations, such as OGT overexpression or OGA inhibition, increase *O-*GlcNAcylation across many proteins and make it difficult to assign phenotypes to modification of a specific protein of interest. Targeted *O-*GlcNAc editing instead aims to install *O-*GlcNAc selectively, enabling functional dissection of protein-specific *O-*GlcNAcylation. POI, protein of interest.

Because *O-*GlcNAcylation occurs on a broad array of proteins, maintaining its homeostasis is critical for normal cellular functions.^5,12^ Consequently, aberrant *O-*GlcNAcylation is a hallmark of numerous pathologies, including cancer, neurodegenerative diseases, and diabetes.^13–15^ However, the promiscuity of OGT means that the biological outcome of *O-*GlcNAcylation is highly context-dependent and protein-specific. A single sugar moiety can dictate a protein’s stability, alter its subcellular localization, or rewire its interactome.^16–18^ Therefore, to truly understand the functional consequences of this PTM in health and disease, it is imperative to study *O-*GlcNAcylation at the level of individual proteins.

Historically, the study of *O-*GlcNAc biology has been severely hindered by a lack of precision tools. Traditional approaches have heavily relied on genetic manipulation (knockout, knockdown, or overexpression) of OGT/OGA, or the use of potent global small-molecule inhibitors.^6,19–27^ While these tools have been foundational, they inherently perturb global *O-*GlcNAc homeostasis, creating confounding effects that make it difficult to define the functions of *O-*GlcNAcylation on individual protein substrates (Figure 1B).^28,29^

Inspired by PROTACs but distinct from their degradative mechanism, we envisioned that chemically induced proximity (CIP) can redirect *O-*GlcNAc transferase activity of OGT toward selected proteins of interest (POIs).^30–36^ In this Account, we describe the development of OGTACs for target-directed *O-*GlcNAc writing and distill four general principles: 1) induced proximity can substitute for native substrate recognition, but only with productive ternary complex conformation; 2) OGT engagement must preserve its catalytic activity; 3) rational selection of target-binding motifs determines the accessible target space of OGTACs; and 4) functional interpretation requires OGTACs to be paired with orthogonal validation of modification sites, target engagement, and downstream phenotypes.

## 2 Chemogenetic Induction of Targeted *O-*GlcNAcylation

Several genetically encoded and macromolecular strategies had shown that OGT activity can be redirected toward selected proteins and enable precise *O-*GlcNAc editing, including nanobody–OGT fusions,^37,38^ dual-specificity RNA aptamers,^39^ optogenetic OGT recruitment,^40–42^ *S*-GlcNAc mimic,^43^ and semisynthetic approaches^44,45^ (Figure 2A). These strategies differ in mechanism, specificity, and experimental accessibility, but they establish an important premise: OGT substrate selection can be rewired when the enzyme and substrate are placed in a favorable spatial context. The question we sought to address was whether an easy-to*-*use, bifunctional small molecule could redirect OGT activity in living cells. Such an approach may provide a synthetically accessible, reversible, and dose-tunable platform for redirecting OGT activity in cellular contexts.

**Figure 2:**
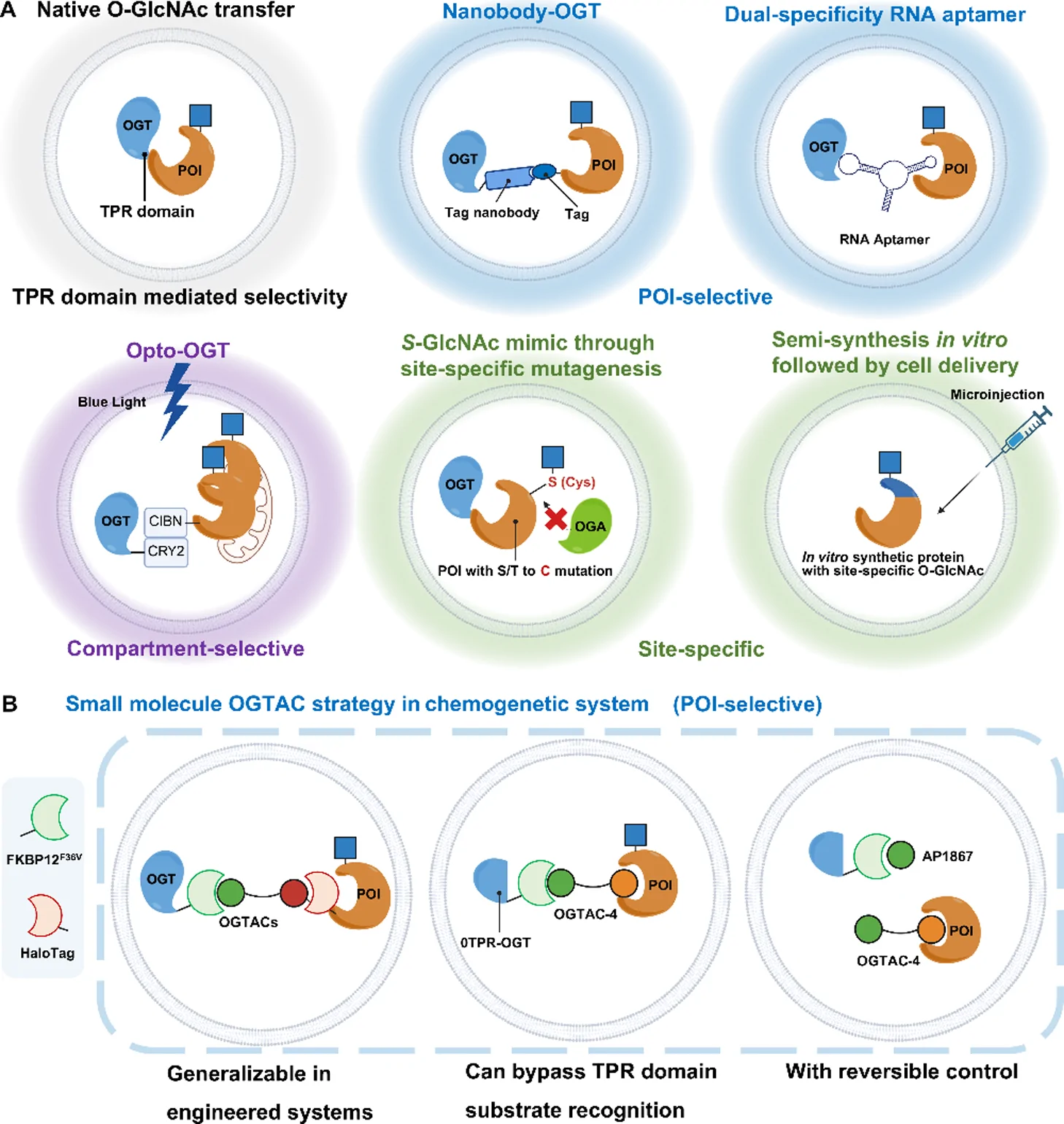
Chemical biology approaches for targeted protein *O-*GlcNAcylation. (A) Comparison of native OGT substrate recognition with representative targeted *O-*GlcNAcylation strategies, including nanobody-OGT fusions, dual-specificity RNA aptamers, opto*-*OGT, *S*-GlcNAc mimic generated by CRISPR-Cas9-mediated Ser/Thr-to-Cys mutagenesis, and semisynthetic approaches. TPR, tetratricopeptide repeats. (B) Chemogenetic OGTACs use bifunctional small molecules to recruit engineered OGT to POIs. This platform demonstrates that induced proximity can redirect FKBP12^F36V^-OGT substrate selectivity, and can be generally applied to different HaloTag-fused POIs, and that the induction effect can be independent of OGT TPR domain. Furthermore, the interaction can be controlled reversibly.

We therefore conceptualized *O-*GlcNAc targeting chimeras (OGTACs) as bifunctional molecules composed of an OGT-recruiting element, a target-recognition motif, and a linker.^1^ To distinguish the effect of proximity from the complications of endogenous target engagement, we first used a chemogenetic system in which OGT was fused to FKBP12^F36V^ (an engineered “bump-and-hole” mutant of human FKBP12 that selectively binds a synthetic ligand^46^) and candidate substrates were fused to HaloTag.^1,47^ Bifunctional molecules containing AP1867 (a synthetic ligand that selectively binds engineered FKBP12^F36V^ protein) and a chloroalkane ligand (for HaloTag) brought the two engineered proteins together.^47–50^ This simplified architecture allowed us to ask whether enforced colocalization was sufficient to increase *O-*GlcNAcylation on a recruited protein.

BRD4 is an epigenetic reader and transcriptional regulator that binds acetylated histones to regulate gene expression. Initial studies in our group showed that BRD4 *O*-GlcNAcylation was readily detectable, establishing BRD4 as a tractable target for subsequent OGTAC activity evaluation. Among the tested molecules, OGTAC-1, which contains the shortest linker, was the most effective inducer of BRD4 *O*-GlcNAcylation and showed rapid modification kinetics.^1^ Notably, the longer-linker analogues OGTAC-2 and OGTAC-3 promoted stronger detectable ternary-complex formation but less efficient *O-*GlcNAcylation. This discrepancy showed that OGTAC output is not dictated simply by ternary-complex abundance, but by the appropriate conformation of the induced complex and its compatibility with *O-*GlcNAc transfer.

After validating the OGTAC effects in a tagged system, we next asked whether the target protein could be engaged through a native protein domain rather than a tag fusion. Replacing the chloroalkane ligand with JQ1 (a small-molecule ligand of bromodomain and extraterminal domain proteins, including BRD4)^51^ generated OGTAC-4, which recruits engineered OGT to the native bromodomain of BRD4. Although detecting the induction effect still required overexpression systems, OGTAC-4 in principle removed the need for a tag on the POI side and introduced ligand-competitive, reversible control (Figure 2B). Furthermore, proteomics followed by site-directed mutagenesis localized OGTAC-1- and OGTAC-4-induced *O*-GlcNAcylation to the S1051–F1224 region of BRD4.^1^ Notably, OGTAC-4 induced detectable *O*-GlcNAcylation over a broader tested concentration range and at lower concentrations than OGTAC-1 under the respective assay conditions.^1^ However, because these systems differ in target-recognition element and binding mode, and because permeability, intracellular concentration, ternary-complex cooperativity, and binding kinetics were not directly measured, the mechanistic basis of this difference remains unresolved. Future studies that integrate quantitative cellular exposure, target-engagement measurements, and matched dose–response analyses will be important for defining structure–activity relationships and improving OGTAC design.

Although OGTAC treatment did not visibly alter total cellular *O*-GlcNAcylation, global immunoblotting may miss subtle pathway-level or site-specific effects. Even low-level overexpression of OGT may increase background *O*-GlcNAcylation and OGTAC treatment could potentially deplete OGT from other important interacting proteins, affect normal signaling, or promote nonphysiological modification events. One strategy to minimize such effects is to use OGT variants with truncated tetratricopeptide repeat (TPR) domains, which reduces native substrate-recognition capacity while preserving catalytic activity when OGT is chemically recruited to a target protein.^52,53^ The ability of truncated OGT constructs to install *O*-GlcNAc under induced-proximity conditions demonstrates that OGTACs can partially bypass native TPR-mediated substrate recognition when the catalytic domain is positioned productively near the substrate (Figure 2B).^1^ This finding does not diminish the importance of the TPR domain in native OGT biology, but instead illustrates a defining feature of CIP-induced targeted *O*-GlcNAc modification. Going forward, more physiologically relevant systems, such as transient recruitment of endogenous OGT, will provide an important benchmark against overexpression-based systems using truncated OGT variants. Global proteomic, transcriptomic, and site-resolved glycoproteomic analyses will help define the physiological relevance of these systems and systematically assess pathway-level perturbations and off-target effects.

## 3 Recruiting OGT without Blocking Catalytic Activity

Broader application in complex cellular systems and *in vivo* models requires OGTAC designs with fewer engineered components. Moving from OGT fusion constructs to fusion-free OGT is therefore essential, but it introduces a central chemical challenge: many high-affinity OGT ligands were developed as inhibitors,^22,54^ and persistent catalytic-site occupancy can suppress the *O-*GlcNAc transfer reaction that OGTACs are intended to promote.^22,54^ For an enzyme-recruiting chimera, binding is useful only if the recruited enzyme remains catalytically competent. This creates a central chemical constraint for OGTAC design: OGT engagement must be compatible with *O-*GlcNAc transfer (Figure 3A).

**Figure 3:**
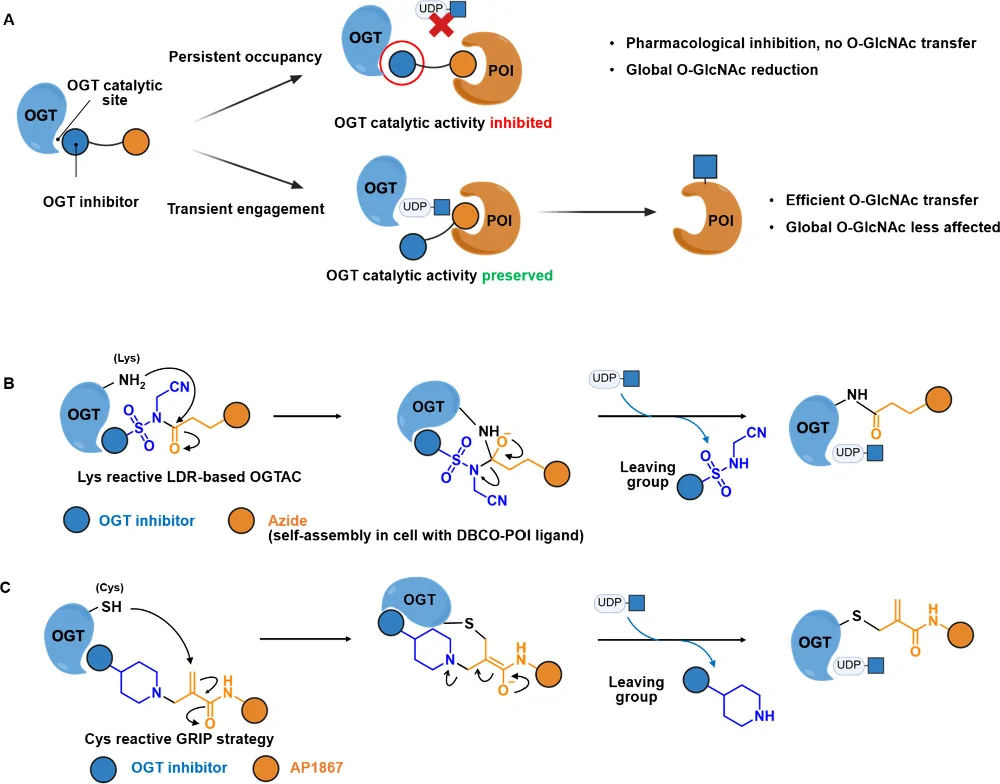
Recruiting OGT through its catalytic domain for targeted *O*-GlcNAcylation. (A) OGT inhibitors can recruit OGT but may block UDP-GlcNAc transfer, preventing productive *O-*GlcNAc installation. Recruitment requires transient engagement that preserves OGT transferase activity. (B) Representative ligand-directed strategies for recruiting OGT through catalytic domain engagement. Lysine-reactive ligand-directed release (LDR) chemistry labels overexpressed fusion-free OGT while releasing the inhibitory recognition element, enabling subsequent bioorthogonal self-assembly with a POI-binding motif. (C) Cysteine-directed GRoup-transfer chimeras for Inducing Proximity (GRIP) provide a complementary route to engage OGT.

We addressed this issue by converting an inhibitor-derived recognition scaffold into a ligand-directed release (LDR) probe.^2^ The minimized quinolinone-6-sulfonamide (Q6S) element guided a reactive *N*-acyl-*N*-alkyl sulfonamide (NASA) group toward OGT, after which the inhibitory recognition element was released (Figure 3B).^55–59^ This strategy produced a covalent OGT handle while reducing persistent active-site inhibition (Figure 3B).^2^ After intracellular self-assembly with a POI-binding motif, the labeled OGT can be recruited to the target protein to promote targeted *O-*GlcNAcylation (Figure 3B).^2^

Our studies with NASA-based OGTACs provide proof of concept that LDR chemistry can induce targeted *O*-GlcNAcylation, while also highlighting the need for further assessment of proteome-wide specificity and endogenous target engagement.^2^ Because NASA exhibits relatively high lysine reactivity compared with more hydrolytically stable transfer groups, the activity and selectivity of these molecules should be established experimentally rather than assumed from design alone.^59–62^ Future lysine targeting LDR-based OGTAC development should therefore benchmark alternative lysine reactive warheads, including more hydrolytically stable transfer groups such as *N*-acyl-*N*-aryl sulfonamides (ArNASAs) and *N*-sulfonyl-*N*-trifluoromethyl ethanamide (SuFA).^59,63^ Furthermore, stability measurements, endogenous target-engagement assays, and global chemoproteomic or proteomic profiling will be essential for evaluating on-target activity, off-target reactivity, and broader glycoproteome-level effects, thereby guiding the design and development of target-specific LDR-based OGTACs that recruit endogenous OGT.^59^

A subsequent preprint describing a group-transfer chimera for inducing proximity (GRIP) strategy for proximity-induced *O*-GlcNAc editing also achieved targeted *O*-GlcNAcylation using LDR, in which methacrylamide was incorporated as a cleavable linker motif.^63^ Whereas our LDR-based OGTAC technology labels lysine residues on OGT, GRIP chemistry targets cysteine residues on OGT to facilitate targeted *O-*GlcNAcylation (Figure 3C).

Together, LDR-based OGTACs and GRIP strategies demonstrate that potent OGT inhibitors can be repurposed as OGT-recruiting binders to enable proximity-induced modulation of *O-*GlcNAcylation. These strategies provide important steps toward fusion-free OGT recruitment, although further work is needed to improve efficiency, target scope, and quantitative understanding in native cellular contexts.

## 4 Rational Selection of the Target-Recognition Motif

Beyond OGT recruitment, the target-recognition motif of an OGTAC determines the accessible proteins or protein complexes. In early chemogenetic systems, tag (e.g., HaloTag) ligands provided a clean and experimentally controlled route to tagged proteins. Small-molecule binders, such as bromodomain ligands, allowed recruitment to ligandable native domains. However, many regulatory proteins, especially transcription factors, lack deep binding pockets but recognize defined nucleic acid motifs. We therefore asked whether a DNA-recognition element could be repurposed as the target-recognition motif of an OGTAC (Figure 4).^3,64,65^

**Figure 4:**
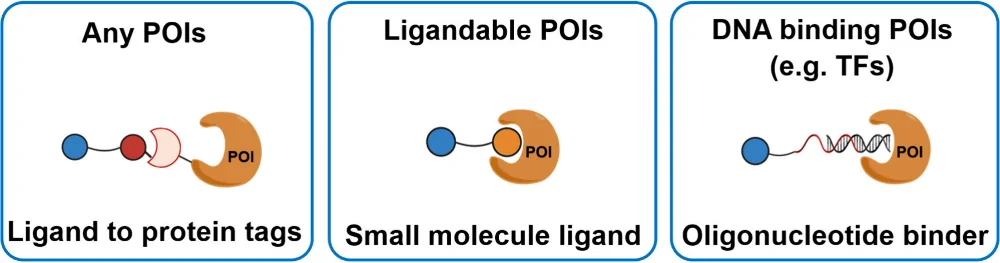
Rational selection of target-recognition motifs. OGTACs share a common bifunctional logic in which an OGT-engaging element is connected to a target-recognition motif, but the identity of this target-recognition motif determines which category of proteins can be reached. Tag ligands provide a controlled entry point for engineered proteins, small-molecule binders enable recruitment to ligandable native domains, and oligonucleotide motifs can enrich DNA-binding protein complexes. In the DNA-based oligo-OGTAC system, the oligonucleotide motif enriches c-Myc-associated transcriptional complexes and supports OGT-dependent modulation of detectable *O-*GlcNAc signals and transcriptional output. Because the DNA motifs can be recognized in broader transcription-factor contexts, this system should be interpreted as target-enriched rather than strictly c-Myc-specific.

As an initial example, we designed DNA-based oligo*-*OGTACs containing E-box motifs intended to engage c-Myc and recruit engineered OGT through an AP1867 handle. The optimized oligo-OGTAC increased c-Myc–OGT proximity and enhanced detectable c-Myc-associated *O-*GlcNAc signals in an OGT-dependent manner, without detectably increasing global *O-*GlcNAc under the tested conditions.^3^

Rather than presenting c-Myc as a solved target, this example establishes a broader design principle: noncanonical recognition motifs can be intentionally incorporated into OGTACs to improve potency and selectivity.^3^ Oligonucleotides and other biomolecular ligands may allow proximity-directed *O-*GlcNAc editing to move beyond the ligandable proteome. At the same time, these recognition motifs require stringent controls to distinguish POI-enriched effects from broader motif- or complex-associated effects.

In summary, the representative OGTAC systems developed to date differ in their OGT-recruitment mode, target-recognition motif, cellular implementation, and degree of site-level resolution. To clarify these distinctions, Table 1 summarizes selected OGTAC designs and their experimental contexts. This comparison highlights a recurring principle: OGTAC activity depends jointly on OGT engagement, target recognition, linker architecture, cellular expression context, and the availability of orthogonal evidence for induced *O*-GlcNAcylation.

**Table 1:** Summary of Representative OGTAC Systems Developed in Our Studies

POI	POI-recognition motif	OGT recruited	OGTAC	Effective concentration/time	*O*-GlcNAc site(s)/region
HaloTag-BRD4	HaloTag ligand	FKBP12^F36V^-OGT	OGTAC-1	100 nM-4 μM/4–24 h	S1051–F1224 region
	JQ1	FKBP12^F36V^-OGT/TPR-truncated OGT	OGTAC-4	3 nM-10 μM/4–24 h	

HaloTag-CK2α	HaloTag ligand	FKBP12^F36V^-OGT/TPR-truncated OGT	OGTAC-1	16–1000 nM/4–24 h	S347
	HaloTag ligand	HA-OGT	LDR-based OGTAC	200 μM/8h; then 0.1–10 μM/16 h^a^	

c-Myc	Oligonucleotide	FKBP12^F36V^-OGT	oligo-OGTAC	0.1–10 μg·mL^–1^/24 h	Remains unresolved

a For the LDR-based OGTAC system, cells were pretreated with 200 μM NASA-based OGT labeling probe for 8 h, followed by treatment with 0.1–10 μM azide–HaloTag ligand for 16 h to enable intracellular self-assembly.

## 5 From Targeted *O-*GlcNAc Writing to Functional Decoding

The central value of OGTACs is that they enable direct, protein-specific perturbation of *O-*GlcNAcylation, allowing *O-*GlcNAc-dependent protein functions to be dissected when paired with existing orthogonal validation methods. Among all classes of *O-*GlcNAc-modified proteins, kinases and transcription factors (TFs) are particularly informative targets because they regulate two major dimensions of cellular physiology.^38,66,67^ Kinases transmit and amplify signaling information through phosphorylation networks, whereas TFs bind to genomic DNA and initiate downstream gene expression. *O-*GlcNAcylation on a single protein of either class can therefore have effects that extend beyond the modified protein itself.

OGTACs are particularly useful for studying such proteins, because pharmacological inhibition cannot easily distinguish direct effects from global changes in *O-*GlcNAcylation. In this way, the OGTAC platform can help address the mechanistic question: what does *O-*GlcNAcylation do when it occurs on a kinase or a TF, rather than across the entire glycoproteome?

### 5.1 Kinases: Decoding PTM Crosstalk, Downstream Signaling, and Protein Turnover

Kinases are attractive targets for targeted *O-*GlcNAc editing because their function is already governed by dense PTM regulation. Phosphorylation controls kinase activity, substrate preference, localization, and stability, and many kinase signaling networks are further shaped by ubiquitination, acetylation, and protein–protein interactions.^38,66,67^ *O-*GlcNAcylation adds another regulatory layer that can intersect with phosphorylation either by modifying nearby residues, altering local structure, changing substrate engagement, or influencing protein turnover.^66^ However, because kinase pathways are highly interconnected, global *O-*GlcNAc perturbation often makes it difficult to determine whether a signaling change originates from modification of the kinase itself or from upstream and downstream network effects.

CK2α provided a useful test case for this problem. CK2α is a pleiotropic kinase with broad signaling roles and prior evidence of *O-*GlcNAc regulation.^68,69^ Using the first-generation chemogenetic OGTAC platform, HaloTag-fused CK2α was recruited to engineered OGT, enabling proximity-dependent *O-*GlcNAcylation (Figure 5A). This system allowed CK2α *O-*GlcNAcylation to be altered in a more focused manner and then connected to site mapping, mutagenesis, stability assays, and downstream phosphorylation readouts.^4^

**Figure 5:**
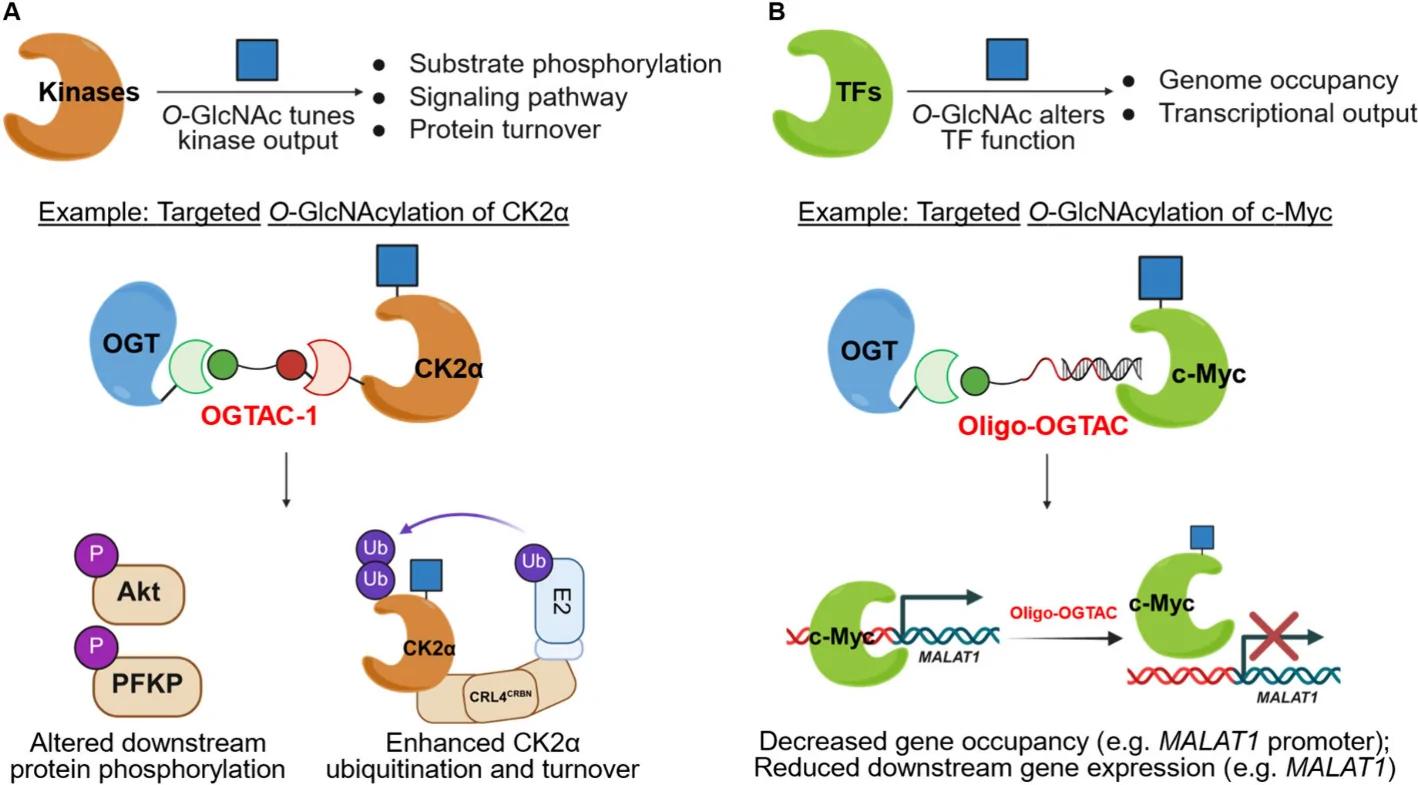
OGTACs enable decoding of functional outputs of kinases and transcription factors (TFs). (A) For kinases, OGTAC-induced *O-*GlcNAcylation can tune the kinase function, especially substrate phosphorylation, pathway signaling, and protein turnover. For instance, targeted CK2α *O-*GlcNAcylation enhances CRL4^CRBN^-associated ubiquitination and CK2α turnover while altering selected downstream phosphorylation events. (B) For TFs, OGTAC-induced *O-*GlcNAcylation can modulate their genome occupancy and downstream transcriptional output. For example, targeted c-Myc O-GlcNAcylation reduced c-Myc occupancy at the MALAT1 promoter and consequently decreased MALAT1 expression.

Chemoenzymatic mass-shift analysis and mutational studies supported Ser347 as the main *O-*GlcNAcylation site.^4^ Mutation of Ser347 attenuated both the detectable *O-*GlcNAc signal and downstream phenotypes, supporting a model in which the observed biological output depends not only on induced proximity but also on the single *O-*GlcNAcylated residue.^4^ Functionally, OGTAC-induced CK2α *O-*GlcNAcylation was linked to ubiquitin–proteasome-dependent turnover.^4^ Cycloheximide chase and proteasome-inhibition experiments showed accelerated CK2α degradation after OGTAC treatment, and co*-*immunoprecipitation and knockdown studies supported enhanced engagement of the CRL4^CRBN^ E3 ligase complex to CK2α.^4^

The same system also revealed that *O-*GlcNAcylation can rewire kinase substrate selectivity.^4^ Before substantial CK2α degradation was observed, OGTAC treatment altered selected downstream phosphorylation events, including Akt Ser129/Ser473 and PFKP Ser386, without causing broad kinase hyperactivation.^4^ Thus, CK2α illustrates how targeted *O-*GlcNAcylation can connect three regulatory layers: local *O-*GlcNAcylation, selective signaling output, and subsequent protein turnover. More broadly, this example suggests that OGTACs can be used to interrogate how *O-*GlcNAcylation reshapes kinase signaling nodes rather than simply increasing global *O-*GlcNAc levels.

### 5.2 Transcription Factors: Probing Transcriptional Activity and Downstream Gene Expression

Transcription factors represent a distinct and important class of *O-*GlcNAc-regulated proteins.^38,66,67^ Unlike kinases, transcription factors generally lack well-defined small molecule binding pockets, and their function usually includes genomic DNA binding and the initiation of downstream gene expression. *O-*GlcNAcylation can influence these processes by altering protein stability, interaction surfaces, or transcriptional activity.^38,66,67,70^ However, transcription factor regulation is especially difficult to interpret using global *O-*GlcNAc perturbation because confounding effects may arise from direct modification of the transcription factor, modification of cofactors, altered chromatin enzymes, or secondary network responses. Complementary to the recent nanobody–OGT system,^38^ our DNA-based oligo-OGTAC targeting c-Myc provides another example harnessing the CIP concept (Figure 5B).^3^ This molecule recruits OGT to c-Myc, increasing c-Myc–OGT proximity and enhancing detectable c-Myc *O-*GlcNAcylation.^3^ At the same time, no detectable increase in global *O-*GlcNAc was observed under the tested conditions.

To connect this local perturbation with transcriptional output, the oligo-OGTAC system was paired with genome-wide CUT&Tag profiling and RT-qPCR. These analyses showed that oligo-OGTAC treatment rewired c-Myc transcriptional activity and was associated with reduced c-Myc occupancy at the downstream gene *MALAT1* locus, and its decreased expression.^3^ This example highlights the potential of targeted *O-*GlcNAc editing to probe transcription-factor functions.

### 5.3 General Lessons from Functional Elucidation of Protein *O*-GlcNAcylation Using OGTACs

Taken together, the kinase and transcription factor examples illustrate two complementary ways in which OGTACs can decode the functional consequences of targeted *O-*GlcNAcylation. OGTACs can reveal not only how local *O-*GlcNAcylation intersects phosphorylation, ubiquitination, signaling output, and protein turnover, but also how *O-*GlcNAcylation influences gene-regulatory events.

Beyond inducing *O-*GlcNAcylation, OGTACs are tools for functional interrogation of protein-specific *O*-GlcNAcylation. OGTACs have the potential to combine localized *O-*GlcNAc installation with orthogonal validation, including site mapping, mutagenesis, proximity assays, protein-stability measurements, phospho- and glycoproteomics or biochemical readouts, and genomic profiling. Together, these validation strategies position OGTACs as powerful tools to resolve protein-directed *O-*GlcNAc functions. Beyond CK2α and c-Myc, many functionally important *O*-GlcNAcylated kinases and transcriptional regulators remain attractive candidates for targeted *O*-GlcNAc modulation, including AMPK, AKT, and other signaling kinases, as well as Sp1, FOXO1, and other stress- or signal-responsive transcription factors.^71^

OGTACs have also been adopted by other groups to probe OGT-dependent biology. For example, OGTAC-2 was recently used to recruit FKBP12^F36V^-OGT to G3BP1-HaloTag under NaAsO2 stress, revealing a non-catalytic role of OGT in regulating G3BP1-associated stress granules.^72^ This example highlights the broader utility of OGTAC-mediated proximity for uncovering both catalytic and noncatalytic functions of OGT.

## 6 Conclusion and Outlook

*O*-GlcNAc biology is difficult to dissect because OGT and OGA are the only two enzymes responsible for writing and erasing *O*-GlcNAc, respectively. As a result, conventional chemical inhibition or genetic perturbation can broadly disrupt *O*-GlcNAc homeostasis and confound target-specific interpretation. To address this challenge, several targeted strategies have been developed, including nanobody–OGT systems, RNA-aptamer-mediated endogenous OGT recruitment, optogenetic approaches, *S*-GlcNAc mimics, and semisynthetic methods. Among CIP-based approaches, nanobody–OGT systems provide genetically encoded target recognition and high specificity, optogenetic tools offer spatiotemporal control, and RNA aptamers enable endogenous OGT recruitment. Of note, these methods often require engineered protein or nucleic-acid components, transfection, or substantial system-specific optimization. Small molecule-based OGTACs therefore offer a complementary CIP strategy for redirecting OGT activity in living cells, with potential advantages in synthetic accessibility, reversibility, dose-dependent tunability, and cellular delivery.

The central lesson from OGTAC development is that OGT can be redirected by chemically induced proximity, but productive *O-*GlcNAc writing requires more than bringing two proteins together. The recruited enzyme must remain catalytically competent, the induced complex must adopt a geometry compatible with transfer, and the target-recognition motifs must be chosen with a clear understanding of their specificity and delivery constraints. These requirements define a broader framework for PTM-inducing chimeras, where the output depends on the compatibility of enzyme engagement, target engagement, and ternary-complex conformation.

Several challenges remain in the targeted protein *O-*GlcNAcylation research field. A central challenge is to transiently recruit endogenous OGT or OGA to modulate target-protein *O*-GlcNAcylation in native cellular systems. Such approaches would better preserve physiological *O*-GlcNAc dynamics while minimizing artificial modifications and externally induced stress responses. Achieving this goal will require improved efficacy and selectivity across different OGT- and OGA-recruitment strategies. LDR-based OGTACs illustrate this challenge clearly: they must recruit OGT efficiently enough to promote productive proximity, while avoiding excessive OGT inhibition that compromises *O-*GlcNAc transfer or perturbs global *O-*GlcNAcylation. Overcoming this trade-off will require systematic optimization of OGT binding affinity, linker length, linker composition, and target-ligand positioning. In parallel, *de novo* high-throughput screening for noninhibitory allosteric OGT binders could expand the OGTAC design space and accelerate their use in native biological contexts. Although OGTAC-1, bearing the shortest PEG2 linker, was the best-performing molecule in our current HaloTag/FKBP12^F36V^ systems, linker optimization will remain critical for future applications involving diverse endogenous targets and distinct modification sites.

A second challenge is defining the structural basis of productive proximity. Ternary-complex formation alone does not necessarily predict glycosylation output, as shown by linker-dependent differences in chemogenetic OGTACs. Future work should combine quantitative modeling with structural and biophysical approaches, including cryo-electron microscopy, X-ray crystallography, NMR spectroscopy, cross-linking mass spectrometry, and computational modeling, to relate ligand affinity, linker architecture, complex geometry, residence time, and OGA-mediated removal to site-specific *O*-GlcNAc installation. Such studies will help identify the OGTAC-induced ternary-complex architectures that enable productive glycosylation.

A third challenge is assigning OGTAC-induced *O*-GlcNAcylation at site-level resolution and relating these sites to physiologically relevant ones. In our studies, CK2α modification was mapped by site-directed mutagenesis to S347, a previously reported physiologically relevant *O*-GlcNAc site, suggesting that OGTACs can enhance modification at an endogenous regulatory site.^4,68^ BRD4, in contrast, showed induced *O*-GlcNAcylation at multiple sites within the S1051–F1224 region by IP–MS, consistent with clustered modification reported in a recent preprint, although the functions of these sites remain elusive.^1,73^ For c-Myc, OGTAC-induced *O*-GlcNAcylation was observed, but the precise site or sites remain to be defined, including whether they overlap with naturally occurring or functionally annotated *O*-GlcNAc sites. Because site mapping remains difficult for low-stoichiometry *O*-GlcNAcylation, especially on low-abundance or intrinsically disordered proteins, future studies should combine proximity characterization assays, site-resolved glycoproteomics, site-directed mutagenesis, and phenotypic functional assays.

Beyond OGTAC-mediated *O*-GlcNAc installation, targeted *O*-GlcNAc removal remains comparatively underexplored. Pioneering approaches, including nanobody/split-OGA systems,^74^ GRIP technology,^63^ and our recently developed de-*O*-GlcNAcylation-targeting chimeras (DOGTACs),^75^ provide initial proof of concept for proximity-directed protein deglycosylation. Future efforts should broaden endogenous target scope while preserving OGA catalytic activity and minimizing disruption of global *O*-GlcNAc homeostasis, ultimately enabling complementary control over the installation and removal of this modification.

Together, these advances reinforce the broader potential of chemically induced proximity for targeted manipulation of *O*-GlcNAcylation. Future in vivo applications of OGTACs will require improved OGT/POI binders and tissue-selective delivery strategies to enable cell-type-specific modulation. Ultimately, OGTACs expand induced proximity beyond targeted degradation, opening a path toward programmable control of protein function, cellular signaling, and disease-relevant biology.
